# Injury activated alveolar progenitors (IAAPs): the underdog of lung repair

**DOI:** 10.1007/s00018-023-04789-6

**Published:** 2023-05-11

**Authors:** Lei Chong, Negah Ahmadvand, Afshin Noori, Yuqing Lv, Chengshui Chen, Saverio Bellusci, Jin-San Zhang

**Affiliations:** 1 Department of Pediatric Respiratory Medicine, National Key Clinical Specialty of Pediatric Respiratory Medicine, Institute of Pediatrics, The Second Affiliated Hospital and Yuying Children’s Hospital of Wenzhou Medical University, Wenzhou, 325027 Zhejiang China; 2 Department of Cell Biology, Duke University School of Medicine, Durham, NC27710 USA; 3 Cardio Pulmonary Institute, Department of Pulmonary and Critical Care Medicine and Infectious Diseases, Universities of Giessen and Marburg Lung Center, Justus-Liebig University Giessen, 35392 Giessen, Germany; 4 Medical Research Center, The First Affiliated Hospital of Wenzhou Medical University, Wenzhou, 325000 Zhejiang China; 5 The Quzhou Affiliated Hospital of Wenzhou Medical University, Quzhou People’s Hospital, Quzhou, 324000 Zhejiang China; 6 Zhejiang Provincial Key Laboratory of Interventional Pulmonology and Department of Pulmonary and Critical Care Medicine, The First Affiliated Hospital of Wenzhou Medical University, Wenzhou, 325000 Zhejiang China; 7 Laboratory of Extracellular Matrix Remodelling, Cardio Pulmonary Institute, Department of Pulmonary and Critical Care Medicine and Infectious Diseases, Universities of Giessen and Marburg Lung Center, Member of the German Lung Center, Justus-Liebig University Giessen, 35392 Giessen, Germany

**Keywords:** Alveolar type 2, Injury-activated alveolar progenitor, Pd-l1, Lung injury repair, Fgfr2b

## Abstract

Alveolar epithelial type II cells (AT2s) together with AT1s constitute the epithelial lining of lung alveoli. In contrast to the large flat AT1s, AT2s are cuboidal and smaller. In addition to surfactant production, AT2s also serve as prime alveolar progenitors in homeostasis and play an important role during regeneration/repair. Based on different lineage tracing strategies in mice and single-cell transcriptomic analysis, recent reports highlight the heterogeneous nature of AT2s. These studies present compelling evidence for the presence of stable or transitory AT2 subpopulations with distinct marker expression, signaling pathway activation and functional properties. Despite demonstrated progenitor potentials of AT2s in maintaining homeostasis, through self-renewal and differentiation to AT1s, the exact identity, full progenitor potential and regulation of these progenitor cells, especially in the context of human diseases remain unclear. We recently identified a novel subset of AT2 progenitors named “Injury-Activated Alveolar Progenitors” (IAAPs), which express low levels of Sftpc, Sftpb, Sftpa1, Fgfr2b and Etv5, but are highly enriched for the expression of the surface receptor programmed cell death-ligand 1 (Pd-l1). IAAPs are quiescent during lung homeostasis but activated upon injury with the potential to proliferate and differentiate into AT2s. Significantly, a similar population of PD-L1 positive cells expressing intermediate levels of SFTPC are found to be expanded in human IPF lungs. We summarize here the current understanding of this newly discovered AT2 progenitor subpopulation and also try to reconcile the relationship between different AT2 stem cell subpopulations regarding their progenitor potential, regulation, and relevance to disease pathogenesis and therapeutic interventions.

## Introduction

**Fig. 1:**
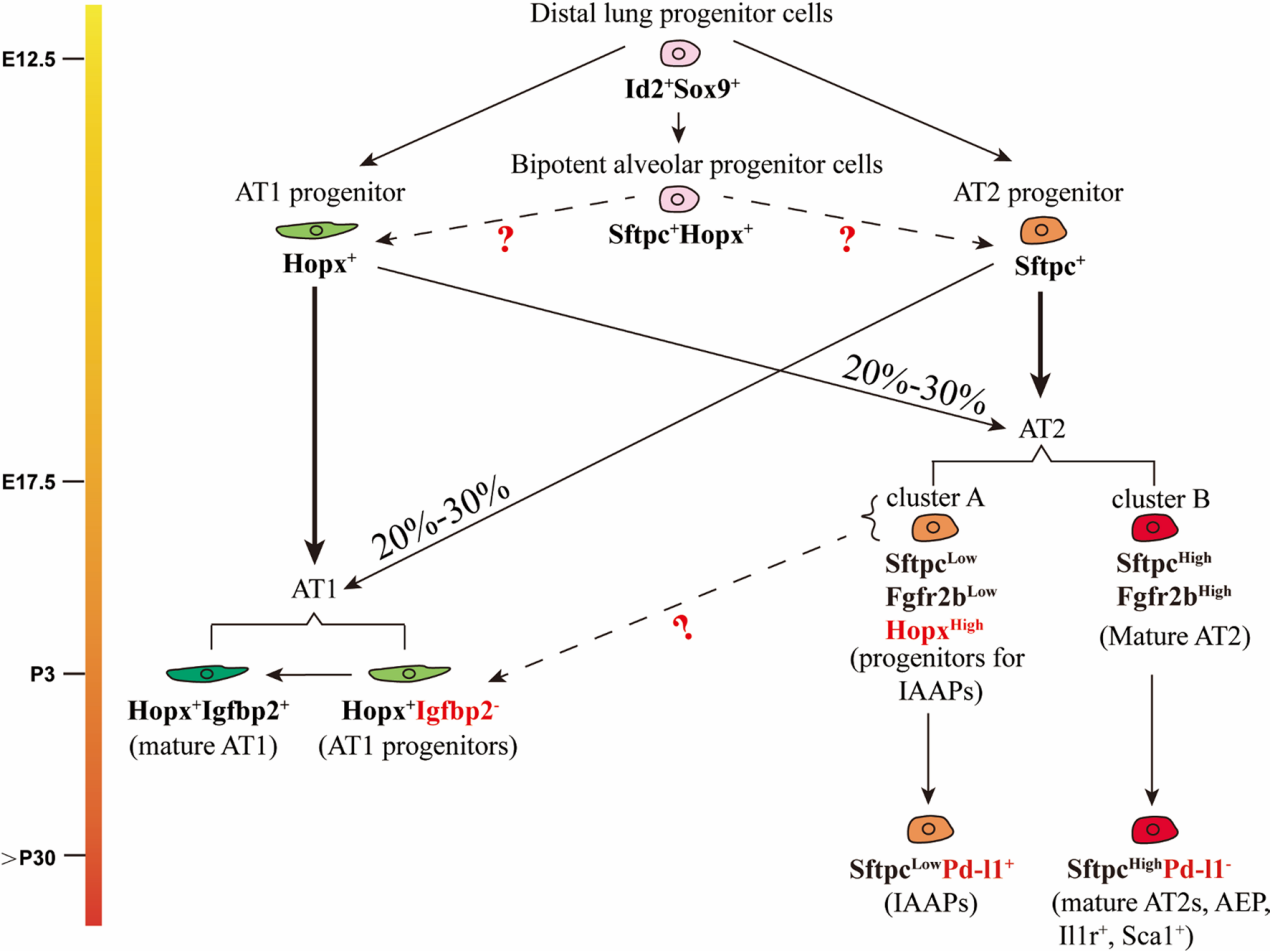
Continuum of AT1 and AT2 formation from lung ontogeny to homeostasis. During early lung development (E12.5), AT1 and AT2 progenitors and bipotent progenitors (BPs) form from distal lung progenitor cells (Id2^+^Sox9^+^). From E13.5 to E17.5, Hopx^+^ AT1 progenitors differentiate into mature AT1 cells. Mature AT1 cells can be classified through the expression of insulin-like growth factor binding protein 2 (Igfbp2). Igfbp2^+^AT1 cells are terminally differentiated cells while Igfbp2^−^AT1s are progenitors for mature AT1s. AT1 progenitors at E14.5 onwards can also contribute to the AT2 lineage. Sftpc^+^ AT2 progenitors differentiate into mature AT2 cells. scRNAseq data indicate that mature AT2 cells can be subdivided into 2 groups (called cluster A and B). Cluster A is Sftpc^Low^, Fgfr2b^Low^ and Hopx^High^ and could represent the progenitors for the IAAPs which express Pd-l1. It remains unclear if cluster A can contribute to the Hopx^+^Igfbp2^−^ AT1 progenitor cells. Cluster B is Sftpc^High^ Fgfr2b^High^ and represents mature AT2s. In this group, AEPs, Il-1r^+^ AT2s and Sca1^+^ AT2s stem cells are present. AT2 progenitors also contribute to the AT1 lineage from E14.5 onwards. The contribution of BPs to the AT1 and AT2 lineage during development is still unclear

Alveoli are the basic structural unit for gas exchange. Alveolar development occurs in the late stage of lung development. Compared to the complex pseudostratified bronchial epithelium, the alveolar epithelium is relatively simple and consists of two cell types; the large flattened AT1s which cover most of the alveolar surface area and provide an effective interface with the microvascular endothelium, and the cuboidal and smaller AT2s, which in addition to producing surfactants, regulating alveolar fluid movement and secreting a variety of antimicrobial peptides to regulate innate immune response. AT2s also serve as a prime source of facultative stem cells during lung regeneration/repair [1, 2]. In this context, the facultative stem cells usually refer to differentiated cells in a resting state that can function as stem cells during repair and regeneration after injury. Both AT1s and AT2s are derived, during lung development, from distal airway progenitor cells which express Inhibitor of differentiation 2 (Id2) and Sex determining region Y—box 9 (Sox9) (Fig. 1) [3–5].

In recent years, two different models of alveolar lineage specification and formation out of these Id2^+^ Sox9^+^ cells have been proposed: the bipotential progenitor model and the early lineage specification model, as illustrated in Fig. 1. The bipotential progenitor model proposes that Id2^+^ Sox9^+^ cells give rise to a population called “bipotential progenitor cells (BPs)” [6]. These cells were found around E16.5 in the mouse, and could self-renew or differentiate into either of the two alveolar epithelial lineages. Based on single-cell transcriptomic analysis conducted at different embryonic timepoints, it was shown that BPs display a gene signature characteristic of both mature AT1s and AT2s. During alveologenesis, BPs have been proposed to downregulate one of the two alveolar epithelial cell signatures, while upregulating the other to become mature alveolar epithelial cells. However, an important limitation of the work supporting this model was that lineage tracing of the BPs was missing; it was unclear what proportion of mature alveolar epithelial cells pass through a BP state.

The second and more recent model of alveolar lineage formation proposes that the majority of mature AT1s and AT2s arise from unipotent (committed), not bipotential, progenitors which are specified as early as E13.5 in the mouse lung [7]. This ‘early lineage specification’ model was supported by single cell transcriptomic analyses along with lineage tracing experiments. In one of these experiments, a dual transgenic mouse line was used to label *Sftpc*-positive and *Hopx*-positive cells at E15.5. These *Sftpc*^+^*Hopx*^+^ cells were considered bipotential, and it was suggested, based on their minor contribution to the more mature AT1 and AT2 cell populations at postnatal day 0 (P0), that BPs play a minor role in alveolar lineage formation. However, given the timepoints chosen in this study to label cells, it remains unclear what proportion of alveolar epithelial progenitors are actually unipotent at E13.5; the significance of the BPs during alveolar lineage formation, therefore, is still to be established.

Recently, we reported that a significant proportion of AT2 and AT1 progenitors during the late pseudoglandular stage of lung development are lineage flexible [8]. In this context, lineage flexibility can be defined as the cross‑lineage contribution of AT1 and AT2 progenitors during early lung development to the opposing lineage, respectively. In support of this process, AT1 and AT2 progenitors were labelled via two tamoxifen intraperitoneal injection (Tam-IP) injections (E14.5 and E15.5), respectively, using *Hopx*^*CreERT2/*+^*; tdTomato*^*flox/flox*^ and *Sftpc*^*CreERT2/*+^*; tdTomato*^*flox/flox*^ transgenic mouse lines. The contribution of lineage-labeled cells to each alveolar epithelial population at E18.5 was assessed. It was demonstrated that around 20–30% of mature pneumocytes derive from lineage-flexible progenitorswhen labeled during mid-pseudoglandular development.

The identity of these progenitors displaying such lineage flexibility remains to be fully clarified. They could arise from either unipotent progenitors which over time acquire the capacity to give rise to the opposite lineage and/or from bipotent progenitor cells which are present already in the early lung expressing both AT1 and AT2 markers. Further studies will have to be conducted to better define the identity of these lineage-flexible progenitors. Interestingly, single cell transcriptomic analysis of whole lung cells captured between E12.5 and P42 led to the identification of a cluster of *Sftpc/Spock2/Hopx*–expressing cells (AT1/AT2) arising at postnatal day 3 (P3). The gene expression signature displayed by this cluster suggests that these cells correspond to a transitional state of AT2 cells similar to *Spock2*^+^*/Axin2*^+^ AT2 cells [9]. Whether these cells arise from the lineage-flexible AT2 cells remain to be clarified.

Besides AT2s, the heterogeneity of the AT1 lineage has also been investigated. AT1 progenitors gradually express insulin-like growth factor binding protein 2 (Igfbp2), a terminal marker of AT1 differentiation. About 62% of Hopx^+^ cells express Igfbp2 at P3 (refer Fig. 1 for details). This percentile is increased to about 95% in the mature lung [10]. The remaining Igfbp2^−^ AT1s, accounting for about 5% of total AT1s, are capable of differentiating into Igfbp2^+^ AT1s and mature AT2s during alveolar regeneration after pneumonectomy thereby indicating their plasticity [10–12]. Of note, upon acute neonatal lung injury (hyperoxia), AT1s reprogram into AT2s, thereby promoting alveolar regeneration. While the ability of AT2s to regenerate AT1s is restricted to the mature lung [11].

## Evidence of AT2 as facultative stem cell

Facultative stem cells are differentiated but quiescent cells, capable of self-renewal or differentiation into other cell types [13]. Since the first radioactive tracing and electron microscopy analysis performed by Evans et al. in 1973, to the study by Barkauskas et al. in 2013, which applied lineage tracing following targeted AT2 ablation and 3D organoid culture, it became clear that AT2s are bona fide facultative stem cells [14–16]. However, in contrast to cells in the skin, intestine and many other tissues with fast homeostatic renewal dynamics, lung alveolar cells display much slower turnover rate, and the less renewing cells are derived from AT2s. In order to study the frequency and spatiotemporal distribution of AT1s renewal by AT2s, Desai et al. used the *LysM-Cre; R26R*^*mTmG*^ mouse to lineage-trace AT2s for up to 16 months. The study demonstrated that AT2s self-renew and generate AT1s in renewal foci deriving from a single founder AT2 cells. Less than 1% of AT1s expressed the AT2-tdTomato lineage tag at 1 month after tamoxifen-based labelling. This percentile is increased to 3.9% and 7.5% at 4 and 16 months, respectively. This indicates that the turnover of alveoli by AT2s is a slow but steady process [17].

The facultative nature of AT2s also raises a series of questions, such as whether all AT2s or only a portion of them have stem cell potential, and whether mechanistically similar proliferative and differentiation processes occur under homeostatic and injury conditions. A series of subsequent explorations further confirmed the heterogeneity of AT2s and stem cell behavior of only subsets of AT2s ^[[[18–24]]]^. For instance, only a portion of AT2s, usually 3–5% of total AT2s, exhibit stem cell properties in the alveolosphere model which co-cultured AT2s with Pdgfra^High^ Cd31/Cd45/Epcam negative resident mesenchymal cells grown in growth factor reduced Matrigel.

Morrisey and Desai groups almost simultaneously and independently reported Wnt-responsive AT2 progenitor/stem cells characterized by the expression of Axin2, however the percentage of Axin2^+^ AT2 cells, in homeostasis, is different from 1% in one study [25] to 20% in the other study [26]. This difference is surprising as both study rely on the use of *Axin2*^*CreERT2*^* driver lines* (*Axin2*^*CreERT2*^*; R26R*^*mTmG*^ and *Axin2*^*creERT2:TdTom*^*; R26R*^*EYFP*^ mice, respectively) and could be attributed to the methodology used to quantify the Axin2^+^ AT2s (FACS vs. immunofluorescence, respectively). Axin2^+^ AT2s (which are also called alveolar epithelial progenitors or AEPs) are evolutionarily conserved alveolar progenitors and showed enriched gene expression profile of lung developmental genes like *Fgfr2, Nkx2.1, Id2, Etv4, Etv5, and Foxa1*. These cells are located close to Pdgfrα-expressing fibroblasts which keep their stemness with the secretion of Wnts. Compared to the Axin2^−^ AT2s, Axin2^+^ AT2s display enhanced self-renewal capabilities in the alveolosphere assays, illustrated by increased colony formation efficiency (around 4% vs. 2%, for AEPs vs bulk AT2s, respectively) and size (around 150 μm vs. 30 μm for AEPs and bulk AT2s, respectively) [25, 26]. In the study reported by the Morrisey group, in context of injury, such as influenza virus (H1N1) infection, Axin2^+^ AT2s (AEPs) which were also described to express Transmembrane 4 superfamily 1 (Tm4sf1) proliferate rapidly under the stimulation of Wnt signals. Interestingly when Wnt signals were withdrawn, AEPs differentiated to AT1s, a process which is instrumental in repairing the alveoli [26]. Previous studies have found that AT2 cells positive for Forkhead box M1 (FoxM1) and Stem cell antigen 1 (Sca1) function as stem cells after infection with Pseudomonas aeruginosa (PA), and differentiate to AT1s to play a repair role under the stimulation of Wnt signaling [27, 28]. How these FoxM1^+^ Sca1^+^ AT2s relate to AEPs is still unclear.

In another study, Katsura et al. detected subsets of AT2 cells which survived influenza-induced injury. These cells are located at proximity to damaged area and proliferate in response to elevated IL-1β and TNFα in the alveolar niche. Interestingly, infiltrating CD45^+^ are found in damaged alveolar regions, suggesting the involvement of immune cells in the epithelial repair process. Moreover, alveolospheres arising from cultured AT2 cells displayed enhanced colony forming efficiency after treatment with interferon α and β, IL-1β and TNFα, and are subjected to regulation by NF-κB signaling activation. This indicates the role of the inflammatory response in AT2s proliferation, however, it is not clear which subset of AT2s are more responsive to inflammatory cytokines [29, 30]. Additionally, Choi et al., introduced a subset of AT2s expressing Il-1r1, which were primed following the secretion of Il-1β from interstitial macrophages during repair. These AT2s acquire a new gene expression profile through the HIF-1α-mediated glycolysis pathway and are called damage-related transient progenitor cells (DATPs). DATPs differentiate to AT1s following bleomycin induced lung injury [29, 30].

Through single-cell sequencing, it was reported that human lung tissues also exhibited a rare cluster of AT2s (called AT2-s) with a distinct transcriptional profile compared to AT2s. These AT2-s selectively expressed components of the WNT signaling (WNT5A, LRP5, CTNNBIP, TCF4, TCF7L2) as well as detoxification genes (CP, GSTA1, CYP4B1). Therefore, it was proposed that AT2-s may be alveolar stem cells that are homologous to Axin2^+^ AT2s in mouse [19]. However, this conclusion may be short lived because many of the other differences in expression between human AT2-s and "bulk" AT2s are not shared when comparing mouse Axin2^+^ AT2s to bulk AT2s [19, 31].

Newly identified alveolar cells, called alveolar cell type 0 (AT0) cells, are related to the alveolar epithelial lineage in the human lung. These cells emerge from AT2s during alveolar repair. AT0 cells are bipotential and co- express *SFTPC*, *SCGB3A2* and different levels of AT1 marker (HTI-56). They give rise to either AT1s or terminal and respiratory bronchioles stem cells (TRB-SCs) depending on their microenvironment. However, it is still unknown whether a subset of AT2 cells are more prone to differentiate to either into AT1s or into TRB-SCs [32]. Therefore, all of these findings support that distinct subsets of the general AT2 population may function as stem cells but also illustrate that our current understanding of these AT2 subsets is still incomplete.

Recently, our team discovered yet another AT2 progenitor subpopulation, which is different from the previously discovered AT2 stem cells [33]. During homeostasis, this subpopulation does not display stem cell activity, but greatly expands after lung injury, filling the compromised AT2 pool. This population appears also to be heterogeneous. An in-depth study of this new subpopulation will certainly complement our knowledge of the composition and function of the different subpopulations composing the AT2 stem cell pool, which will be the focus of this review.

## Identification of the injury activated alveolar progenitors (IAAPs)

Through lineage tracing of tdTomato^+^ cells in the lungs of *Sftpc*^*CreERT2/*+^*; tdTomato*^*flox/flox*^ mice, we found that tdTomato^+^ cells can be divided into two subpopulations, one with low tdTomato level (Tom^Low^ AT2s), and the other with high tdTomato level (Tom^High^ AT2s). Tom^High^ AT2s account for around 80% of lineage-traced AT2s, whereas Tom^Low^ AT2s account for the remaining 20%. The ATAC-seq analysis also confirms that they are two distinct populations with different chromatin configuration. Tom^Low^ AT2s express lower levels of AT2 differentiation markers such as *Sftpc*, *Sftpb*, *Sftpa1*, *Fgfr2b* and *Etv5* compared to Tom^High^ AT2s, and may represent a group of immature AT2s [33]. Moreover, Tom^Low^ AT2s are different from the AT2 stem cell subgroups mentioned above (AEPs, Sca-1^+^ AT2s and Il-1r^+^ AT2s), not only because those AT2 stem cell subgroups express high levels of AT2 differentiation markers, but also because Tom^Low^ AT2s express a low level of *Axin2* (while expressing *Tm4sf1*). Moreover, Tom^Low^ AT2s do not have any role in maintaining the steady-state in the adult lung and are only activated under damage stimulation. For these reasons these cells were called “Injury Activated Alveolar Progenitors” or IAAPs. IAAPs are neither part of lineage negative epithelial progenitor (LNEP)/distal airway stem cells (DASCs) nor part of bronchoalveolar stem cells (BASCs) as LNEP/DASCs are negative for Sftpc and BASCs express high levels of the AT2 marker Sftpc and the club cell marker, Scgb1a1, while they locate in the respiratory epithelium and do not display high levels of Scgb1a1 [34–36]. Therefore, IAAPs may represent a novel subset of quiescent and immature AT2 stem cells that is distinct from the more mature AT2 stem cells.

Screening of the top 100 differential expressed genes between IAAPs and Tom^High^ AT2s (containing mostly the mature AT2s with no or limited stem cell capabilities as well as the mature AT2 stem cells such as the AEPs and Sca1^+^ AT2s) led to the identification of several surface markers expressed at higher level in IAAPs, including programmed cell death-ligand 1 (Pd-l1, also named Cd274), a cell surface molecule associated with immunosuppression, Cd33, an adhesion protein expressed at the surface of myeloid cells [33] and Cd300lf as a regulator of immune response [37]. Data mining of recently published single-cell sequencing data from normal adult human lung cells further confirmed the existence of PD-L1^+^ AT2s [19, 33]. Intriguingly, PD-L1^+^ AT2s sub-cluster displays low levels of ETV5, SFTPC and AXIN2 but high level of TM4SF1. TM4SF1 is an epithelial cancer stem cell membrane protein, which is also expressed by AEPs. The differences between IAAPs, AEPs, Sca1^+^AT2s and Il-1r^+^ AT2s are summarized in Table 1.

**Table 1:** Comparison of IAAPs with mature AT2 stem cells

	IAAPs	AEPs	Sca1^+^AT2s	Il-1r^+^ AT2s
Differentiation status	Immature	Mature	Mature	Mature
Status of activation	PNX and bleomycin injury	Homeostasis and influenza infection	PA infection	Bleomycin injury
Markers	Low levels of AT2 differentiation markers (*Sftpc*, *Sftpb*, *Sftpa1*, *Fgfr2b* and *Etv5*) High levels of *Pd-l1* and *Tm4sf1*	High levels of AT2 differentiation markers (*Sftpc*, *Sftpb*, *Sftpa1*, *Fgfr2b* and *Etv5*), Wnt target gene (*Axin2*) and epithelial cancer stem cell membrane protein (*Tm4sf1*)	High levels of AT2 differentiation markers (*Sftpc*, *Sftpb*, *Sftpa1*, *Fgfr2b* and *Etv5*), stem cell antigen (Sca1) and FoxM1	High levels of AT2 differentiation markers (*Sftpc*, *Sftpb*, *Sftpa1*, *Fgfr2b* and *Etv5*) and *Il-1r*
Stem cell property# in homeostasis	No	Yes	Not clear	Not clear
Stem cell property in repair	Yes	Yes	Yes	Yes
Potential functions	Replenishing AT2 stem cells pool Rescue the loss of mature AT2	Maintaining lung homeostasis Promoting alveolar regeneration	Promoting alveolar regeneration	Promoting alveolar regeneration
References	Ahmadvand et al. [33] Ahmadvand et al. [43] Ahmadvand et al. [42]	Nabhan et al. [25] Zacharias et al. [26]	Liu et al. [28] Liu et al. [27]	Choi et al. [29] Strunz et al. [71]

^#^Assessed using alveolospheres

PNX pneumonectomy, PA Pseudomonas aeruginosa, Tm4sf1 Transmembrane 4 superfamily 1, Pd-l1 Programmed death ligand-1, CD274 Il-1r: Interleukin-1 receptor, Sca-1 Stem cell antigen 1, FoxM1 Forkhead box M1

## A small parenthesis on the use of tomato as a reporter for Cre expression

Our discovery that the level of tomato expression could be used to discriminate between two subpopulations within the AT2 lineage using the *Sftpc*^*CreERT2/*+^*; tdTom*^*flox/flox*^ mice was initially surprising [33]. This difference was observed even when only one copy of *tdTom*^*flox*^ was used (*Sftpc*^*CreERT2/*+^*; tdTom*^*flox/*+^) ruling out that this difference was due to one versus two copies of the *Rosa26*^*LoxP−STOP−LoxP−tdTomato*^ allele recombined in the context of *Sftpc*^*CreERT2/*+^*; tdTom*^*flox/flox*^ mice. As full recombination of this allele was observed both in IAAPs and AT2s, this led us to hypothesize that the difference was instead the result of differential expression of tdTomato from the *Rosa26* promoter per se in the IAAPs vs. AT2s. Indeed, ATAC-seq analysis indicated a more closed chromatin configuration at the *Rosa26* locus in IAAPs vs. AT2s. This phenomenon may be unique to the AT2 lineage or shared by other lineages and careful work has to be carried out on the characterization of tomato intensity to tease out the possibility of capturing distinct lineages. Another important consequence of this differential chromatin configuration is that tomato intensity may potentially be used to monitor the differentiation process of the IAAPs towards the AT2s.

## Possible function of IAAPs

Since IAAPs belong to an immature AT2 stem cell subgroup, their function is likely different from that of the previously discovered mature AT2 stem cells. By 3D co-culture of IAAPs or Tom^High^ AT2s with Sca1^+^ resident mesenchymal cells (rMCs, defined as Cd31/Cd45/Epcam triple negative), respectively, we found that Tom^High^ AT2s form alveolosphere, while IAAPs exhibit a very weak ability to promote organoid formation. Thus, Tom^High^ AT2s contain mature AT2 stem cells [33]. Then, what is the function of IAAPs? The pneumonectomy (PNX) model in mice, through surgical removal of the left lobe, triggers the process of compensatory growth in the remaining right lobes, with a particularly strong response of the accessory lobe. When PNX and Sham surgeries were carried out on *Sftpc*^*CreERT2/*+^*; tdTom*^*flox/flox*^ mice and tamoxifen was administered before the operation to label the IAAPs and AT2s. The robust compensatory growth of the remaining lobes is associated with increased proliferation of AT2s, visible as early as day 5 following PNX [38]. Such an increase is not seen in the Sham operated mice. Lung Epcam^+^ cells account for around 70% of lineage-labeled AT2 cells (either IAAPs or mature AT2s) with the rest being AT1s and bronchial epithelial cells. Surprisingly, analysis at day 7 post-surgery showed that the ratio of IAAPs over Epcam^+^ cells were more than doubled in PNX vs. Sham. While the ratio of mature AT2s over Epcam^+^ cells trended towards a decrease. Furthermore, transcriptional profiling of IAAPs after fluorescence-activated cell sorting (FACS) revealed an increase of *Fgfr2b*, *Etv5*, *Sftpc*, *Cyclin D1 (Ccnd1)*, *Cyclin D2 (Ccnd2)* and *Ki67* expression in PNX compared to the Sham. Overall, these data indicate that IAAPs are activated and proliferate to replenish the mature AT2s in the context of lung regeneration. This strongly suggests that the increase in the mature AT2s observed upon PNX mainly arises from the IAAPs, but not the pre-existing mature AT2s. Subsequent analysis through in vitro culture of precision cut lung slices (PCLS), we found that while Tom^High^ AT2s were massively depleted, IAAPs proliferated. Interestingly, the fluorescence intensity of tdTomato in these cells was gradually increased suggesting their differentiation towards mature AT2s [33].

Further analysis of *Sftpc*^*CreERT2/*+^*; tdTom*^*flox/flox*^ mice with flow cytometry also showed that the percentile of IAAPs over Epcam^+^ cells expanded significantly following bleomycin injury while the percentile of AT2s over Epcam^+^ cells decreased, demonstrating that AT2s represent the main alveolar epithelial target upon bleomycin injury [39]. The percentile of IAAPs increased gradually after bleomycin induction, peaked at day 16 (fibrosis period), then decreased gradually and recovered to the initial level at day 60 (resolution period). On the contrary, the number of Tom^High^ AT2 (mature AT2) decreased to the lowest level on day 16 and returned progressively to normal level on day 60. The inverse correlation between the percentile of IAAPs and AT2s following bleomycin injury suggests that IAAPs may represent an AT2 stem cell pool contributing to replenish the dying AT2s after lung injury [39]. Additionally, the surviving Tom^High^ AT2s at day 16 may contain the AEPs, which will then proliferate and contribute to the restoration of lung homeostasis. Further investigation is required to fully delineate the nature of the survival cells in the Tom^High^ AT2s pool.

Altogether, these results demonstrated that IAAPs are activated only upon injury and that AT2s, which contains the AEPs and other mature AT2 stem cells is at the best not changed in the PNX model or even decreased in the bleomycin model during the first 16 days following injury, thereby raising important questions on the proposed privileged role played by the AEPs upon injury [39].

**Fig. 2:**
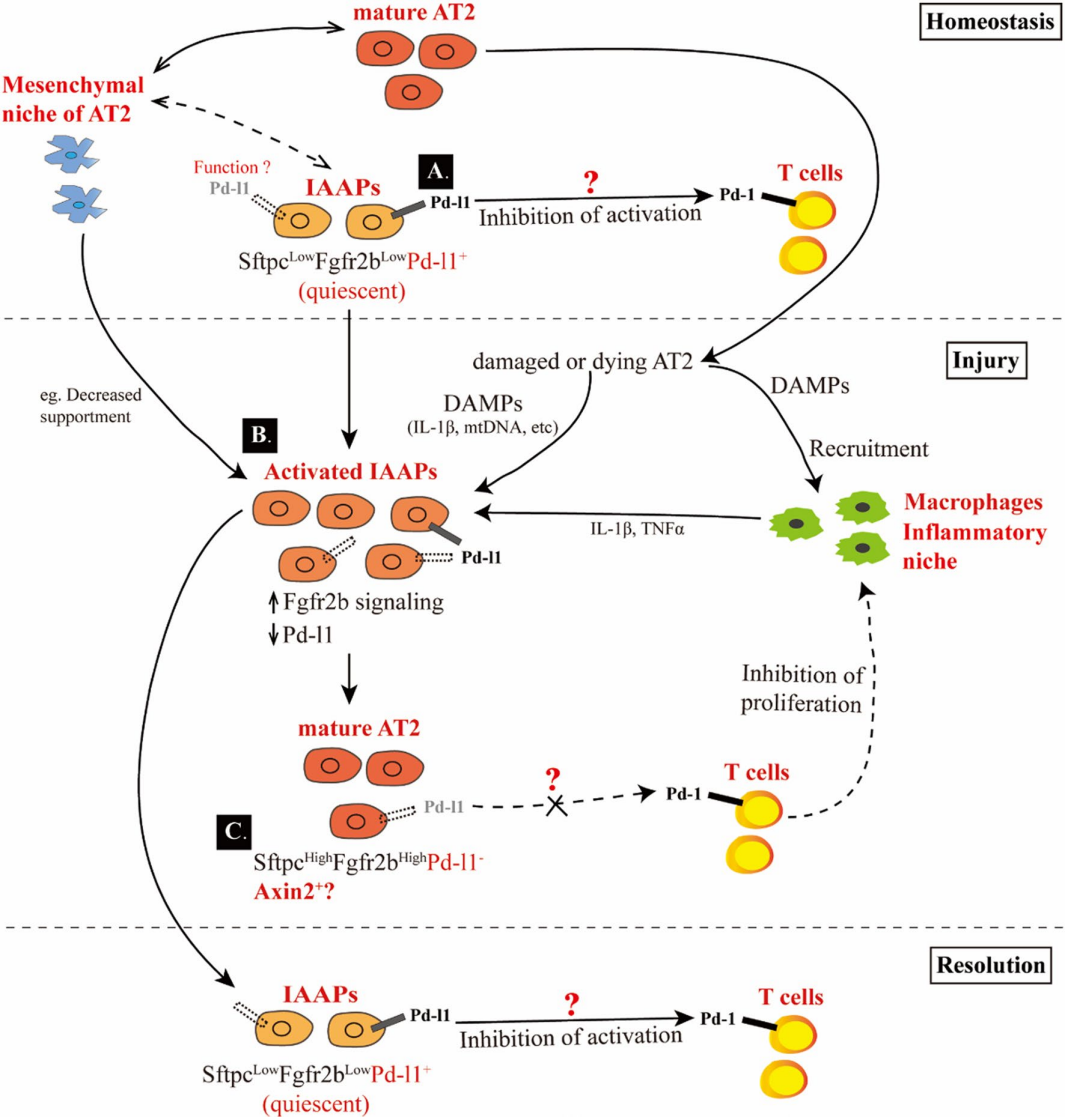
Possible function of Pd-l1 in IAAPs. In homeostasis, IAAPs are quiescent and do not significantly interact with the resident mesenchymal niche for mature AT2s. Around 50% of the IAAPs express Pd-l1. The function of Pd-l1^−^ IAAPs is still unclear. We propose that Pd-l1/Pd-1 signaling inhibits T cell activation, thereby keeping the inflammatory signals low. Mature AT2s interact with the resident mesenchymal niche which is essential for their survival. After injury, mature AT2s cells are dying and release damage activated molecular patterns (DAMPs) such as Il-1 which act on the macrophages for their recruitment and activation and on IAAPs for their proliferation. Inflammatory signals from the macrophages such as Il-1 and Tnfa also contribute to the proliferation of the IAAPs. IAAPs are also interact with the mesenchymal niche to receive survival/proliferative signals such as Fgfs. Activated/proliferative IAAPs progressively differentiate into Pd-l1^−^ mature AT2s to replenish the impaired mature AT2 pool. These Pd-l1^−^ AT2s also re-enforce the inflammatory niche. During resolution, activated Pd-l1^Low^ IAAPs give rise to Pd-l1^High^ quiescent IAAPs which mitigate inflammation through Pd-l1/Pd-1 signaling in macrophages

Through database mining and examination of lungs from IPF patients, we and others found that the percentile of PD-L1^+^ AT2s (similar to mouse IAAPs) over EPCAM was markedly increased in IPF lungs compared to that of the dornors. There was a significant shift of the transcriptome in IPF IAAPs compared to AT2s, including lower AT2 signaling and dysregulation of gene expression related to cell proliferation in IPF patients [40–42]. It also appears that these cells are stalled in their transition to fully mature AT2s. The reasons behind this defect are still unclear and could be related to the high level of inflammatory signals present in diseased lungs which were previously proposed to prevent the differentiation of the DATPs into mature AT1s [29]. Therefore, we speculate that IAAPs serve as progenitors for mature AT2 cells. It is still unclear if IAAPs can also differentiate directly into AT1s, thereby bypassing the previously described transient DATP state (Fig. 2).

## Revisiting the initial demonstration that mature AT2s are stem cells: are the IAAPs the elephant in the room?

The seminal paper establishing the role of AT2s at large as stem cells capable of self-renewal and differentiation towards AT2 and AT1 cells was based on the use of a transgenic mouse model targeting DTA expression in AT2s while at the same time labeling them with tomato (*Sftpc*^*CreERT/*+^*: R26*^*loxP−STOP−Loxp−DTA; loxP−STOP−LoxP−tdTomato*^) [16]. Upon tamoxifen-mediated Cre nuclear translocation in AT2s, the STOP codon at the *Rosa26* locus is being removed allowing the expression of both tdTomato and DTA. In theory, as both tdTomato and DTA are co-expressed, these cells should undergo apoptosis and therefore no lineage traced cells should be observed. So how is it that some lineage- traced cells not only survived but expanded in a clonal fashion? This study attributed this to “chance”, as only one of the two *Rosa26* allele containing the tdTomato but not the DTA was recombined. However, our recent results with the IAAPs allow to propose an alternative, and perhaps a more plausible explanation. What is likely observed in this experiment is the dual labeling of the IAAPs and the AT2s. IAAPs have a less opened chromatin configuration of the *Rosa26* locus thereby allowing very low expression, if any of DTA. It is therefore likely that these IAAPs survived while AT2s, with a high level of DTA were more efficiently eliminated. What is observed in this context therefore could be the clonal expansion of the surviving IAAPs. Another intriguing observation is that the IAAPs appear to develop mechanisms of resistance to deleterious genetic manipulation. For example, *Fgfr2b* deletion in both AT2s and IAAPs leads to cell death. However, the surviving IAAPs manage to prevent the deletion of the *Fgfr2b* allele via a mechanism that remains to be identified [43]. A similar situation could therefore take place in the context of the *R26R *^*loxP−STOP−LoxP−DTA*^ allele. Although, in theory, everything is in place for the recombination of this allele but survival mechanisms (which we propose are mechanisms of resistance) are taking place to prevent the expression of DTA. From an alveolar epithelium standpoint, the IAAPs may represent the last resort to repair the distal lung by functioning as a fail-safe mechanism of self-protection. A similar logic is observed for cancer stem cells which develop ingenious countermeasure to escape chemotherapy.

## Following the IAAPs during injury

The potential events associated with IAAPs activation following injury are illustrated Fig. 2. Located on the luminal surface of the alveoli, epithelial cells are more sensitive to injury or infection resulting in their death. Recently, a number of studies have demonstrated that dysfunctional mature AT2s are the driver of chronic lung injury such as pulmonary fibrosis [44, 45]. Dysfunctional or dying mature AT2s may release damage-associated molecular patterns (DAMPs), triggering pro-inflammatory pathways and Th2 polarizing cytokines, which then initiate the activation of macrophages or maturation and recruitment of other immune cells [46].

As canonical DAMPs, Il-1 family can function in the inflammatory niche to enhance alveolar regeneration [29, 30, 47]. Il-1 has been shown to directly act on mature AT2 stem cells to trigger their proliferation [29]. In the future, it will be important to investigate the potential activating role of Il-1 and other cytokines on IAAPs. We propose that, upon injury or infection, the surviving IAAPs may receive the DAMPs signals from damaged or dying AT2, leading to their activation and expansion. During their differentiation towards the mature AT2s, activated IAAPs may lose distinct molecular marker such as Pd-l1, and acquire Wnt-target genes, such as Axin2 (See Fig. 2C). Future studies using the *Sftpc*^*CreERT2/*+^*; Pd-l1*^*DreERT2*^ double recombinase mouse line to specifically lineage trace Pd-l1^+^Sftpc^+^ IAAPs will be instrumental to study the activation, fate and function of IAAPs.

## Potential roles of Pd-l1 signaling in IAAPs

To the best of our knowledge, the Pd-l1/Pd-1 pathway is an important immune checkpoint in tumor immunotherapy as Pd-l1 displays immunosuppressive activity. When it binds to its receptor Pd-1, expressed on the surface of T or B cells, it inhibits their proliferation. Therefore, in human, anti-PD-L1 treatment can reduce the immune escape of tumor cells and enhance the effect of anti-tumor therapy [48]. However, Pd-l1 may have completely different effects in different diseases or when expressed in different cells. Several recent studies have reported that Pd-l1 expression in lung fibroblasts increases in pulmonary fibrosis and is secreted into exosomes to inhibit the proliferation of T cells and promote the proliferation and migration of fibroblasts. Therefore, it has been proposed that inhibiting the expression of Pd-l1 in lung fibroblasts may improve the process of pulmonary fibrosis [49–51]. Interestingly, the expression of PD-1, the receptor for PD-L1, is up-regulated in IPF lymphocytes, and the PD-1^+^CD4^+^ T cells display reduced proliferative capacity and increased transforming growth factor–β (TGF-β) expression. Both bleomycin administration to *Pd-1*^*−/−*^ mice or use of antibody against PD-L1 demonstrated significantly reduced fibrosis upon loss of PD-L1 expression compared to controls [52]. However, another study on human mesenchymal stem cells (MSCs) found that blocking PD-L1 expression in these cells decreased the efficacy of MSCs in treating pulmonary fibrosis [53]. Other studies in the context of cancer have found that PD-L1 can promote the transformation of hepatic stellate cells into myofibroblasts, accelerating tumorigenesis. Targeting PD-L1 in hepatic stellate cells can selectively inhibit the occurrence of liver cancer [54]. However, increasing the expression of PD-L1 in hepatocytes reduced the liver injury of non-alcoholic fatty liver disease [55]. Knockdown of *Pd-l1* or *Pd-1* gene can also reduce the activity of vascular endothelial cells, enhance the tight junctions of endothelial cells, and significantly improve the survival rate of mice suffering from acute respiratory distress syndrome (ARDS) caused by hemorrhagic shock [56].

IAAPs’s chromatin is more accessible for genes relating to the innate and adaptive immune system [33], suggesting their interaction with the immune cells under such circumstance is fundamentally different from mature AT2s. PD-L1 could therefore play an instrumental role in IAAPs´ stem cell function, since previous studies demonstrated the function of PD-L1 in regulation of cell proliferation in cancerous cells. For instance, Fang et. al, interestingly, found that PD-L1 regulates cell cycle entry in leukemia-initiating cells (LICs) as *PD-L1*-null LICs displayed cell cycle arrest and decreased cell proliferation. Moreover, cell cycle regulators such as P16, P21, Cyclin D2, and CDK6 were significantly regulated in *PD-L1* knock-down cells [57]. Similarly, other studies illustrated the regulation of pancreatic cancerous proliferation through cell cycle- related genes and JNK phosphorylation following PD-L1 overexpression [58]. These suggest the function of PD-L1 in controlling cell cycle entry. Further research is required to study whether similar mechanisms play a role in IAAPs progenitor behavior and how different are these mechanisms from the ones promoting the cells to become cancerous. Another function of Pd-l1 expression in IAAPs might be their protection as privileged progenitor cells from the immune system, similar to what has been shown to protect the hematopoietic stem cells, the stem cells in the hair follicles, and Lgr5^+^ intestinal stem cells [59–61].

Our discovery that Pd-l1 is a molecular marker of IAAPs also raises a number of interesting possibilities (see Fig. 2A). For example, whether Pd-l1 is related to the innate immune response of AT2s, whether it can be wrapped by extracellular vesicles to be released in the blood circulation, or whether IAAPs bind to T cells that infiltrated into the alveoli through the Pd-l1/Pd-1 pathway to promote T cell suppression during lung homeostasis. Interestingly, by losing Pd-l1 expression upon injury, IAAPs may no longer suppress T cells, which are then capable, as part of the inflammatory niche, to trigger the proliferation and differentiation of AT2 stem cells towards the AT1 lineage via Il-1. Inhibiting Pd-l1 in IAAPs may therefore impact positively the regeneration of AT2s. This possibility needs to be further investigated.

## Role of Fgf10/Fgfr2b signaling pathway on IAAPs

In view of the key role played by fibroblast growth factor 10 (Fgf10)/Fgf receptor 2b (Fgfr2b) signaling in lung development, alveolar regeneration and repair, we further investigated the impact of Fgf10/Fgfr2b signaling pathway on IAAPs [62–65]. We found that overexpression of Fgf10 or treatment of recombinant FGF10 (rFGF10) significantly improved the degree of pulmonary fibrosis in bleomycin-injured mice, whether administered simultaneously or at day or day 14 after injury, by promoting the active proliferation of IAAPs [39, 66].

Fgfr2b is the main receptor for Fgf10. In the lung, Fgfr2b restricts AT2 cell fate during alveolar lineage formation and is needed for AT2 survival postnatally [67–69]. An accepted concept in the repair field is that the mechanisms involved recapitulate ontogeny. What occurs to the IAAPs during repair is a good illustration of this principle as the expression of *Fgfr2b* and its downstream factor *Etv5* have been found upregulated in proliferative IAAPs upon injury. These cells have been called “activated IAAPs” (see Fig. 2B). This observation suggests that Fgfr2b signaling, an AT2-specific developmental signaling pathway, is reactivated [43]. Interestingly, Fgfr2b signaling has been proposed to be dispensable for AT2 homeostasis and alveolar repair [68, 70]. However, far from being dispensable, our recent study demonstrated that specific inactivation of the *Fgfr2b* gene in AT2s leads to apoptosis of both AT2s and IAAPs. However, the resulting morphological changes in the mutant lungs were not obvious, suggesting that there must be compensatory mechanisms at play. Further analysis revealed that surviving IAAPs escaped *Fgfr2b* deletion through a mechanism that remains to be identified. These cells were therefore termed “resistant IAAPs” or RIAAPs. We propose that RIAAPs are amplified and differentiate into mature AT2s (we called these cells “differentiated AT2 arising from RIAAPs” or DRIAAP). Subsequently, as DRIAAPs acquire high *Sftpc* expression, the corresponding level of Cre recombinase expression which is under the control of the *Sftpc* promoter is also enhanced, leading to *Fgfr2b* deletion in DRIAAPs. Loss of *Fgfr2b* expression leads to apoptosis thereby creating a constant cycle of proliferative and apoptotic alveolar epithelial cells.

Altogether, this leads to the establishment of a novel proliferation/apoptosis loop in mutant lungs allowing the maintenance of a constant number of alveolar epithelial cells needed for proper lung function [43].

## Open questions and future directions

Although the AT2 lineage has been a major topic of investigation over the years, its study still allows to make significant discoveries offering new insight and opportunities to reconsider some of the dogmas in the field of lung regeneration. For example, how can we reconcile in vivo and in vitro observations about the mature AT2s? While the in vitro studies clearly show that mature AT2s contain stem cells capable of self-renewal and differentiation, the in vivo data, however, clearly show that most of the action in terms of proliferation is taking place in the IAAPs and not the mature AT2s. In addition, it is still not clear whether AEPs, Sca1^+^AT2s and IL-1r^+^AT2s co-exist as distinct, separate subpopulations or if they are part of a continuum within a given differentiation process.

Recently, several papers described "intermediate cells" in AT2 to AT1 transition, which were given by different groups various names, such as Alveolar Differentiation Intermediate (Krt8^+^ADI) [71], Pre-Alveolar Type-1 Transitional Cell State (PATS) [72] or Damage-Associated Transition Progenitors (DATPs) [29]. Strunz et al. found that approximately half of the alveolar Krt8^+^ alveolar differentiation intermediate (ADI) cells were derived from either *Sftpc*^*CreERT2*^ or Sox2^*CreERT2*^ lineage-labelled cells in the bleomycin model. It is proposed that these cells differentiate from elite progenitors belonging to the mature AT2s pool, namely the Tm4sf1^+^ Axin2^+^ AEPs. It remains to be resolved whether IAAPs differentiate into mature AT2s through a Tm4sf1^+^ Axin2^+^ AEP intermediate or directly to AT1s via the transient Krt8^+^ADI cell state. The answers to these important questions will require further investigation.

Another important question that needs to be addressed is whether IAAPs belong to a distinct AT2 sub-population, or represent a transient AT2 cell state. Usually, a transient cell state arises from a stable population of cells in response to injury. As IAAPs represents a group of immature and quiescent lineage-traced Sftpc-positive cells consistently detected during homeostasis, this observation alone would argue that IAAPs constitutes a cell population on their own, distinct from mature AT2s. Obviously, only lineage tracing of the IAAPs, combined with injury models and scRNAseq, will be able to address their capacity to give rise to mature AT2s and AT1. Such approaches will also be instrumental to further define their heterogeneity, which is already suggested by the fact that only half of IAAPs express PD-L1. Another intriguing possibility is whether AT2s can differentiate into IAAPs after injury. This will require the identification of AT2 markers which are not expressed by IAAPs. So far, the use of the *Sftpc*^*CreERT2*^ mice does not allow to answer to this important question as SFTPC is expressed in both AT2s and IAAPs.

The regulatory effect of FGF10 or other target drugs on different AT2 subsets and their therapeutic application to enhance the repair process are also worthy of further study. Furthermore, what is the role of Pd-l1 in IAAPs? Is this just a marker for these cells or does it play an active role in maintaining their function? Promoting or blocking Pd-l1 expression in the context of lung diseases may also be important for the precision therapy of lung diseases. Using dual recombinase approach to specifically label the IAAPs in combination with single cell RNA/ATAC sequencing and spatial transcriptomic in the context of lung injury and regeneration will provide valuable and informative data on the IAAPs with the aim of expanding our knowledge of this new subpopulation of AT2 progenitor cells.

## Data Availability

Not applicable.
